# 以维泊妥珠单抗为基础方案治疗中枢神经系统累及的难治性弥漫大B细胞淋巴瘤1例报告并文献复习

**DOI:** 10.3760/cma.j.cn121090-20240119-00035

**Published:** 2024-09

**Authors:** 洁菲 白, 茹 冯, 婷 王, 旭 李, 龙 钱, 江涛 李, 春丽 张, 辉 刘

**Affiliations:** 1 北京医院血液内科，国家老年医学中心，中国医学科学院老年医学研究院，北京 100730 Department of Hematology, Beijing Hospital, National Center of Gerontology, Institute of Geriatric Medicine, Chinese Academy of Medical Sciences, Beijing 100730, China; 2 北京医院核医学科，国家老年医学中心，中国医学科学院老年医学研究院，北京 100730 Department of Nuclear Medicine, Beijing Hospital, National Center of Gerontology, Institute of Geriatric Medicine, Chinese Academy of Medical Sciences, Beijing 100730, China

## Abstract

维泊妥珠单抗（Pola）是一种靶向CD79b的新型抗体药物偶联物，临床试验显示在初诊和复发/难治的弥漫大B细胞淋巴瘤（DLBCL）中均有效。本文回顾性分析了北京医院1例脊髓受累的难治性继发性中枢神经系统淋巴瘤患者的临床特点及诊治过程，并进行相关文献复习。患者79岁，诊断为DLBCL，累及髂骨、骶骨、脊髓、神经根，IPI评分5分，MSKCC评分高风险，老年综合评估（IACA评分）2分，为不适合强化疗组。一线治疗应用无化疗方案联合放疗，患者病情进展，二线治疗应用以Pola为基础的方案，患者获得完全缓解，提示Pola可能作为累及中枢神经系统的DLBCL患者的治疗选择。

## Abstract (en)

Polatuzumab vedotin（Pola）is a novel antibody-drug conjugate targeting CD79b, which has been shown to be effective in treating newly diagnosed and relapsed/refractory diffuse large B cell lymphoma（DLBCL）during clinical trials. This study aims to conduct a retrospective analysis of the clinical characteristics, diagnosis, and treatment of a patient with refractory secondary central nervous system lymphoma at Beijing Hospital, alongside a review of relevant literature. This study included a 79-year-old female patient who was diagnosed with DLBCL affecting the ilium, sacrum, spinal cord, and nerve roots and had an IPI score of 5 and a high-risk score according to MSKCC. She showed a geriatric comprehensive assessment（IACA）score of 2, which was categorized under the unfit group. Her initial treatment comprised chemo-free therapy and radiotherapy, followed by progression. In the second-line treatment, a Pola-based regimen was applied, and the patient achieved a complete response, suggesting that this regimen may be a therapeutic option for patients with DLBCL involving the central nervous system.

累及中枢神经系统的弥漫大B细胞淋巴瘤（DLBCL）也称为继发性中枢神经系统淋巴瘤，在DLBCL中的发生率为4％～9％[1]–[3]，中位总生存（OS）期为3.9～28个月[3]–[5]。对于年轻、肾功能良好、体能状态佳的患者推荐应用MATRix（甲氨蝶呤+阿糖胞苷+塞替派+利妥昔单抗）与R-ICE（利妥昔单抗+异环磷酰胺+卡铂+依托泊苷）交替方案或MR-CHOP（甲氨蝶呤+利妥昔单抗+环磷酰胺+表阿霉素+长春新碱+泼尼松）方案续贯自体造血干细胞移植，而对于高龄、不耐受化疗的患者建议应用减低剂量的甲氨蝶呤、布鲁顿酪氨酸激酶抑制剂、来那度胺、放疗或嵌合抗原受体T细胞治疗等[6]–[8]。维泊妥珠单抗（Pola）是一种靶向CD79b的新型抗体药物偶联物，临床试验显示在初诊和复发/难治的DLBCL中均有效，且不良反应可控[9]–[11]，但Pola的关键临床试验均排除了中枢神经系统受累的患者，目前国内外尚无Pola在中枢神经系统淋巴瘤中应用的报道。近期本中心采用以Pola为基础的方案成功治疗1例难治性继发性中枢神经系统淋巴瘤患者，报告如下并进行文献复习。

## 病例资料

患者，女，79岁，2023年3月患者无诱因出现腰背部疼痛，无发热、盗汗、体重明显降低等，腰椎MRI示：L4-S4椎体及双侧髂骨异常改变，髂骨前和椎管内硬膜外软组织肿块。此后患者疼痛症状加重，VAS评分8分，需服用吗啡缓释片对症止痛治疗，并出现进行性下肢肌力降低，伴麻木，以右侧为主，小便失禁。2023年4月下肢肌力3～4级，伴疼痛、活动受限，卧床状态（ECOG评分4分），行骶骨病变穿刺活检示：DLBCL，活化B细胞型。免疫组化：CD3（−），CD20（+），CD10（−），Bcl-6（+），Bcl-2（−），Mum-1（个别+），c-Myc（30％+），Ki-67（80％+），PAX5（+），CD38（弱+），CD138（−）。下肢深静脉超声：双侧小腿肌间静脉血栓形成，服用艾多沙班30 mg每日一次，为进一步诊治就诊于我院。入院后完善相关检查：乳酸脱氢酶（LDH）608 U/L，白蛋白（ALB）32 g/L，肌酐（Cr）50 µmol/L，肿瘤组织二代测序：TP53突变66％，B2M突变53.7％，CXCR5突变42.5％，CXCR4突变17.2％，FAS突变25.9％，分子分型为A53亚型。PET-CT：骶骨及骶前-椎管内代谢活性增高的肿块，累及腰骶段脊髓及神经根，较大横截面约10.6 cm×5.4 cm，SUV_max_＝22.6，腰1～3椎管内及相应双侧椎间孔处的神经根放射性摄取不均匀增高，SUV_max_＝7.6（图1A）。因患者无法配合，未行腰椎穿刺术。诊断为DLBCL ⅣA期伴大包块，累及髂骨、骶骨、脊髓、神经根，IPI评分5分，CNS-IPI预后评分5分，纪念斯隆-凯特琳癌症中心（MSKCC）评分高风险。老年综合评估IACA评分量表2分，为不适合强化疗组。入院后患者出现泌尿系统感染，予抗感染治疗后好转。2023年4月开始无化疗方案（ZRD方案），具体为泽布替尼160 mg每日2次，利妥昔单抗375 mg/m^2^第0天，地塞米松10 mg第1～5天，治疗1个疗程。治疗中患者腰背部疼痛逐渐加重，伴谵妄，1个疗程后CT：骶前软组织肿块，范围约12.4 cm×6.8 cm，骶骨、双侧髂骨多发低密度影。考虑疗效欠佳，更换治疗方案为ZR2D方案联合放疗，即在原方案基础上加用来那度胺10 mg第1～21天，联合放疗（部位：骶尾骨、腰1～5椎体、椎管、骶前软组织肿块），总剂量40 Gy。治疗2个疗程后，2023年7月5日（中期评估）PET-CT：颈6～胸12椎管内代谢活性增高的肿块，新出现，SUV_max_＝46.7，累及脊髓及神经根。原骶前至骶管内代谢活性增高的肿块较前缩小，代谢活性减低，范围9.4 cm×5.2 cm×10.4 cm，SUV_max_＝8.8；原腰1～3椎管及相应神经根处代谢活性增高灶此次未见（图1B），考虑疾病进展。2023年7月更换为Pola-RD方案，具体为Pola 90 mg第1天，利妥昔单抗375 mg/m^2^第1天，地塞米松10 mg，第1～5天。治疗3个疗程后（2023年9月）再次评估PET-CT：原颈6～胸12椎管内代谢活性增高灶基本消失。原骶前及骶管内代谢活性增高的肿块较前稍缩小，原其内局灶代谢活性增高灶消失，SUV_max_＝3.9，考虑完全缓解（CR）（图1C）。治疗期间不良反应为中性粒细胞缺乏（中性粒细胞最低值为0.16×10^9^/L）伴发热、泌尿系统感染，尿培养示酵母菌、肺炎克雷伯菌，予氟康唑、美罗培南抗感染治疗后症状好转。此后患者因经济原因未再治疗原发病，2024年1月PET-CT评估：未见新发病灶，Deauville评分3分，椎管内未见代谢活性增高灶；原骶骨前方软组织影范围缩小，代谢稍高，程度大致同前，考虑持续CR（图1D）。目前仍在规律随访中，无进展生存（PFS）期（2023年7月至2024年1月）6个月，OS期9.5个月。

**图1:**
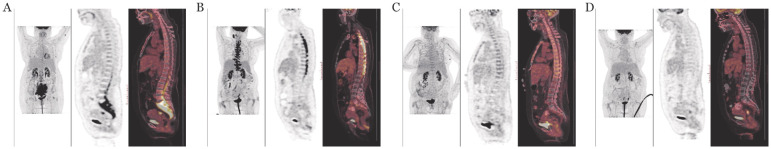
弥漫大B细胞淋巴瘤患者的PET/CT结果 **A** 治疗前；**B** 中期评估；**C** 以维泊妥珠单抗为基础方案治疗后；**D** 2024年1月随访

## 讨论及文献复习

继发性中枢神经系统淋巴瘤病变部位不同，患者可出现不同的症状和体征，常见的部位为柔脑膜、脑实质、脊髓、颅神经或脊神经根，其中脊髓内播散是继发性中枢神经系统淋巴瘤的罕见表现，仅见于小样本病例报告[5],[12]。系统性淋巴瘤的脊髓受累可来自血行播散或柔脑膜淋巴瘤直接侵犯[13]。常见症状为截瘫、背痛、感觉丧失和痉挛等[12]。本例患者起病时的症状为疼痛、下肢无力、膀胱功能障碍等，PET-CT可见骶骨破坏、骶骨旁软组织肿块、神经根和脊髓受累。

针对中枢神经系统淋巴瘤的MSKCC评分系统根据年龄和Karnofsky体能状态（KPS）将患者分为3个风险组：低风险（年龄<50岁）、中等风险（年龄≥50岁且KPS≥70分）、高风险（年龄≥50岁且KPS<70分）[14]。一项多中心回顾性研究纳入92例2000年至2010年治疗的继发性中枢神经系统淋巴瘤患者，72％的患者接受了系统性化疗（含甲氨蝶呤或阿糖胞苷），29％的患者接受了造血干细胞移植，中位OS期为7个月，MSKCC低风险、中等风险和高风险患者的中位OS期分别为16个月、9个月和2个月[15]。研究显示，初次治疗未获得CR的继发性中枢神经系统淋巴瘤患者中位OS期仅为2.3个月[5]。此例患者MSKCC评分为高风险，予ZR2D方案联合放疗后，病情仍在进展，故预后极差。

对于继发性中枢神经系统受累的患者，治疗目标是持久控制全身和中枢神经系统淋巴瘤、提高生存质量、延长PFS期和OS期。本例患者年龄79岁，入院时存在症状性泌尿系统感染，老年综合评估为不适合强化疗组，与家属沟通后选择无化疗方案ZRD，1个疗程后疼痛症状加重，疾病评估提示疾病稳定，调整治疗方案为ZR2D联合放疗，再次评估提示疾病进展。后续可供选择的治疗方案少，预后极差。

Pola是靶向CD79b的新型抗体药物偶联物，可与淋巴瘤细胞表面靶抗原结合，复合物被迅速内吞并分解，效应分子单甲基澳瑞他汀E（MMAE）释放后阻滞细胞分裂并导致凋亡，产生的旁观效应可进一步杀伤周围其他肿瘤细胞[16]–[17]。GO29365是一项多中心、开放标签的以复发/难治DLBCL为研究对象的临床试验，试验组接受Pola-BR方案（Pola+苯达莫司汀+利妥昔单抗，40例），对照组接受BR方案（苯达莫司汀+利妥昔单抗，40例）。研究显示，试验组总反应率为45％，CR率为40％；而对照组总反应率为17.5％，CR率为17.5％。中位随访22.3个月，试验组和对照组的中位PFS期分别为9.5个月、3.7个月，中位OS期分别为12.4个月、4.7个月。两组的不良反应可控[10]。POLARIX研究以初诊DLBCL为研究对象，其中试验组接受Pola-R-CHP方案（141例），对照组接受R-CHOP方案（140例），试验组和对照组的2年PFS率分别为74.2％和66.5％，两组的安全性相当[11]。但临床研究均排除了中枢神经系统受累的患者，目前尚无Pola治疗中枢神经系统淋巴瘤的报道。Pola作为抗体偶联药物类大分子药物，理论上透过血脑屏障的作用较弱，但由于淋巴瘤累及中枢神经系统的患者有不同程度的血脑屏障破坏，使Pola可能穿过血脑屏障，通过测定脑脊液内Pola的浓度能显示Pola是否能进入中枢神经系统。另外，Pola与利妥昔单抗作用于淋巴瘤的不同靶点，其联合应用在DLBCL的治疗中有协同作用。

本病例是脊髓受累的继发性中枢神经系统淋巴瘤，较罕见，一线经无化疗方案联合放疗病情进展，二线选择以Pola为基础的方案，新加入的药物仅有Pola，经治疗后患者疾病获得CR，提示Pola可以作为中枢神经系统受累DLBCL患者的治疗选择。
